# Supplementary material for: Analysis of the glyco-code in pancreatic ductal adenocarcinoma identifies glycan-mediated immune regulatory circuits
Source: Commun Biol. 2022 Jan 11;5:41. doi: 10.1038/s42003-021-02934-0 (PMC8752754; doi:10.1038/s42003-021-02934-0)
Supplement: Supplementary file 2 — Supplementary Information [file 42003_2021_2934_MOESM2_ESM.pdf]

Supplementary Information

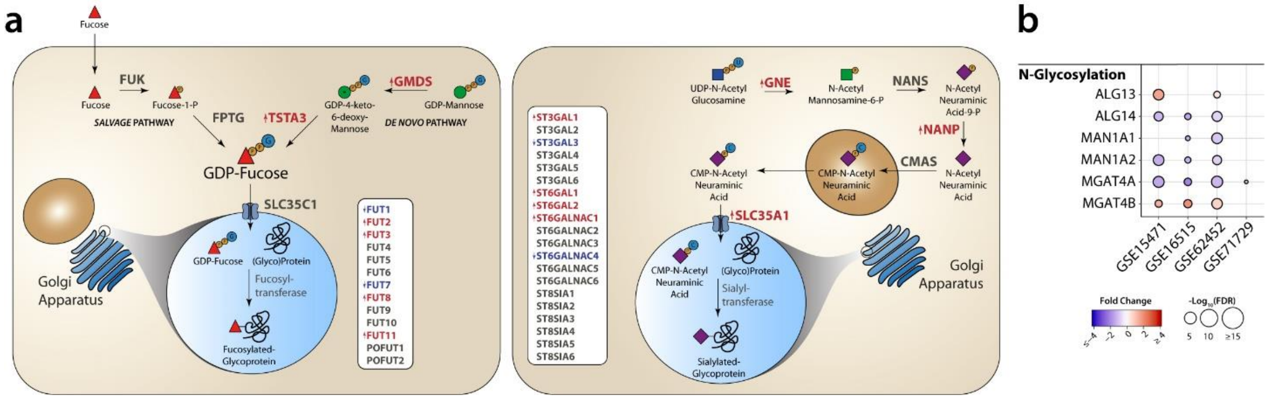

Figure S1. Fucosylation and Sialylation pathways in PDAC. Related to Figure 1. a) Graphic representation of the pathways for the synthesis of the fucose- and sialic acid- donors. b) Changes in N-Glycosylation in PDAC tissue are not as pronounced as changes in the glyco-code.

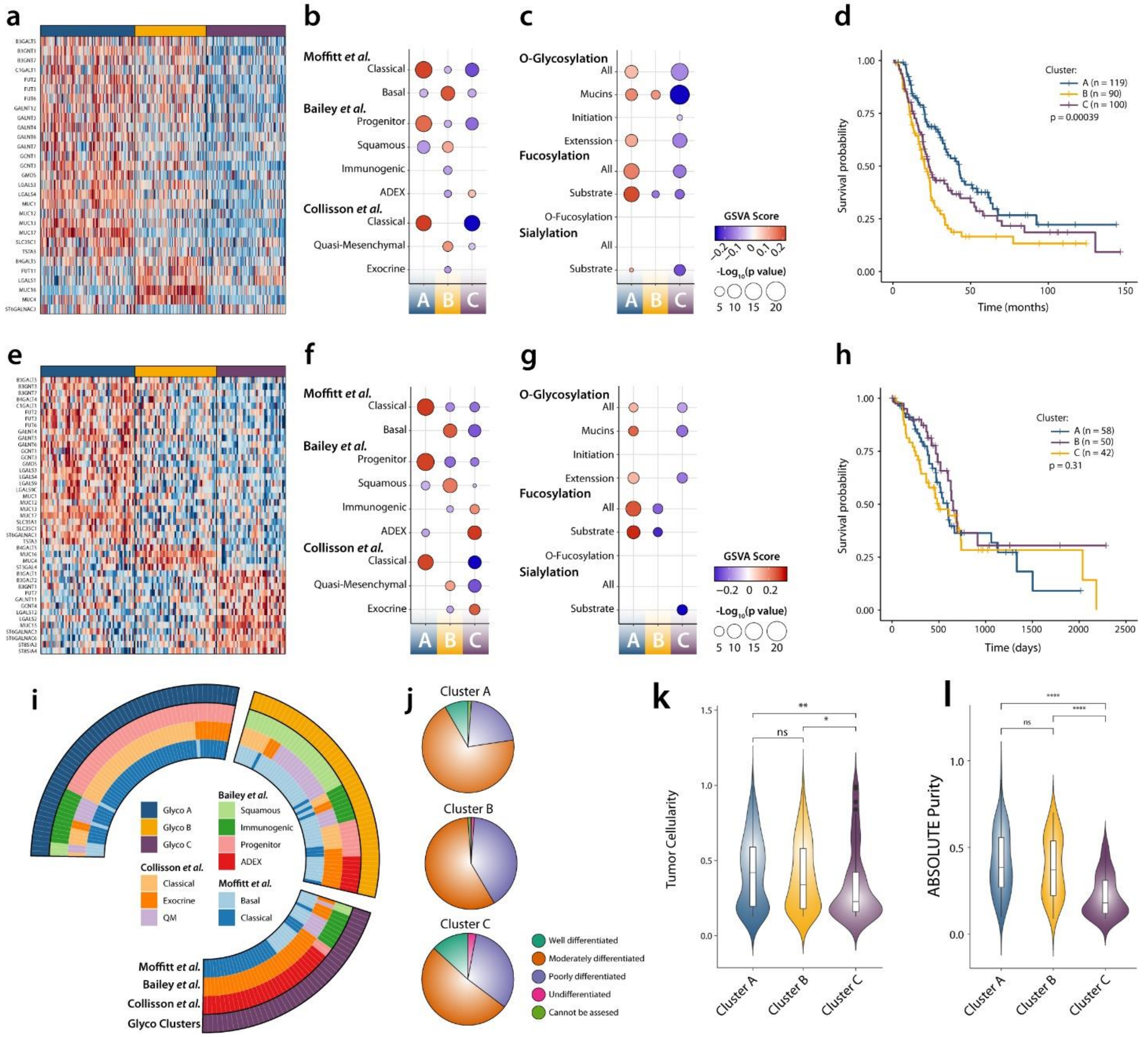

Figure S2. Characterization of glycosylation driven Subtypes. Related to Figure 2. The 3 Clusters can be also found in the validation data sets: E-MTAB-6134 (a-d) and TCGA-PAAD (e-h). For each data set we show: heatmap of differential expressed glyco-code related genes (a, e), GSVA enrichment score for gene sets associated with subtypes previously described (b, f) and glycosylation pathways (c, g) and survival analysis (d, h). i) Circular heatmap showing the association of glycosylation subtypes present in the TCGA cohort and molecular subtypes described by Bailey *et al*, Collisson *et al* and Moffitt *et al*. j) Tumor differentiation grade in the different glyco-code associated subtypes of the TCGA data set. k) Tumor cellularity in subtypes of the ICGC data set. l) ABSOLUTE purity in each subtype of the TCGA data set. Pairwise comparisons: Mann-Whitney test *p ≤ 0.05, **p ≤ 0.01, ***p ≤ 0.001, ****p ≤ 0.0001.

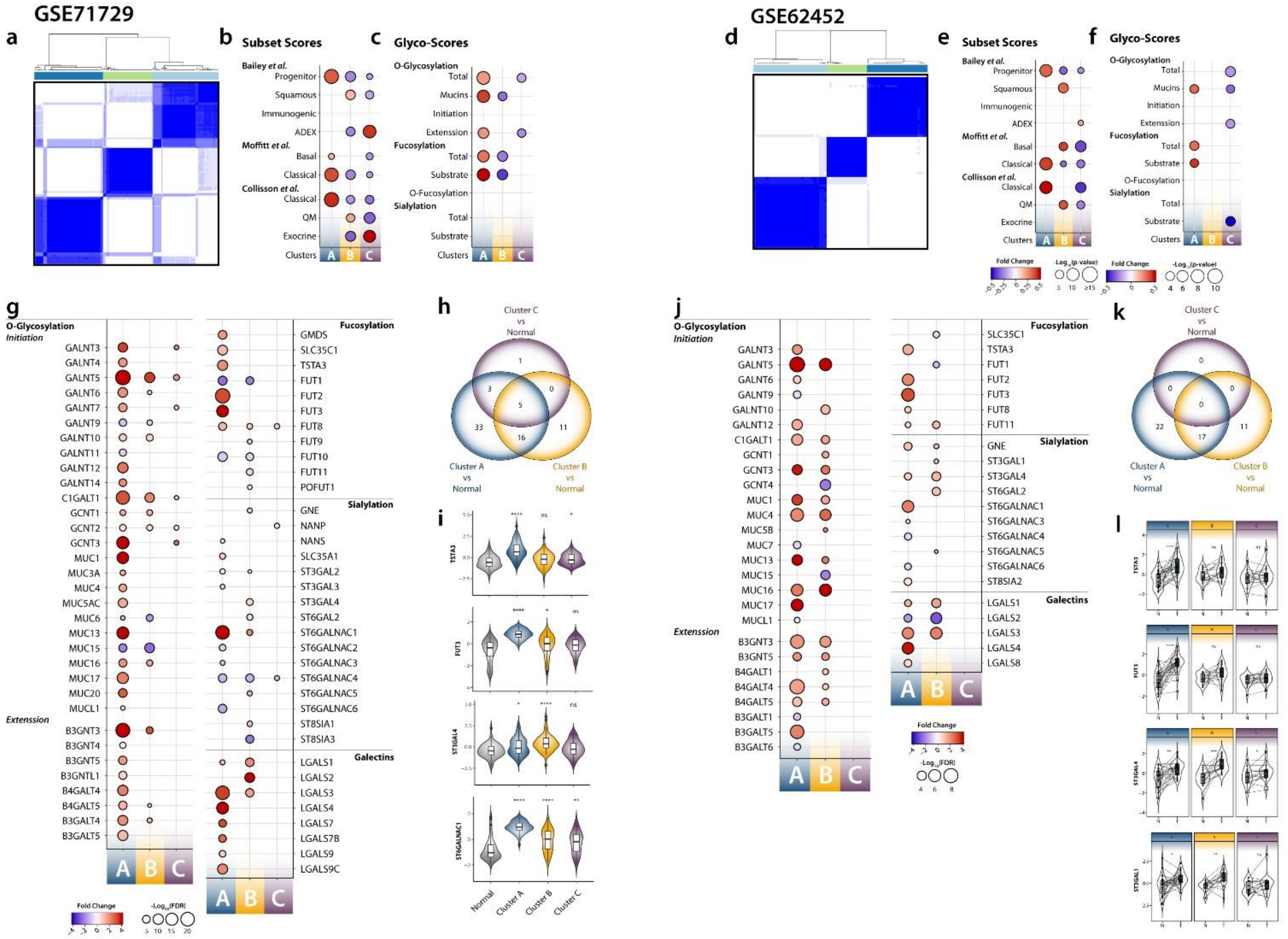

Figure S3. Differentially expressed genes between the glycosylation Clusters and normal tissue. Related to Figure 2. The *Basal* and *Fucosylated* subtypes can also be found in other two datasets: GSE71729 (a-c) and GSE62452 (d-f). For each data set are shown: consensus-heatmap (a,d), GSVA enrichment score for gene sets associated with subtypes previously described (b,e) and glycosylation pathways (c,f). The package *limma* was used to determine the differential expression of glyco-code genes between the different clusters present in tumor and normal tissue using an unpaired (GSE71729, g-i) or paired (GSE62452, j-l) analysis. Bubble plot and Venn diagrams for differentially expressed genes in each cluster (cut off – FDR < 0.05, g-h, j-f). Violin plots of selected fucosylated and sialylated genes (i,l). Pairwise comparisons of each Cluster against Normal Tissue: Mann-Whitney test *p ≤ 0.05, **p ≤ 0.01, ***p ≤ 0.001, ****p ≤ 0.0001.

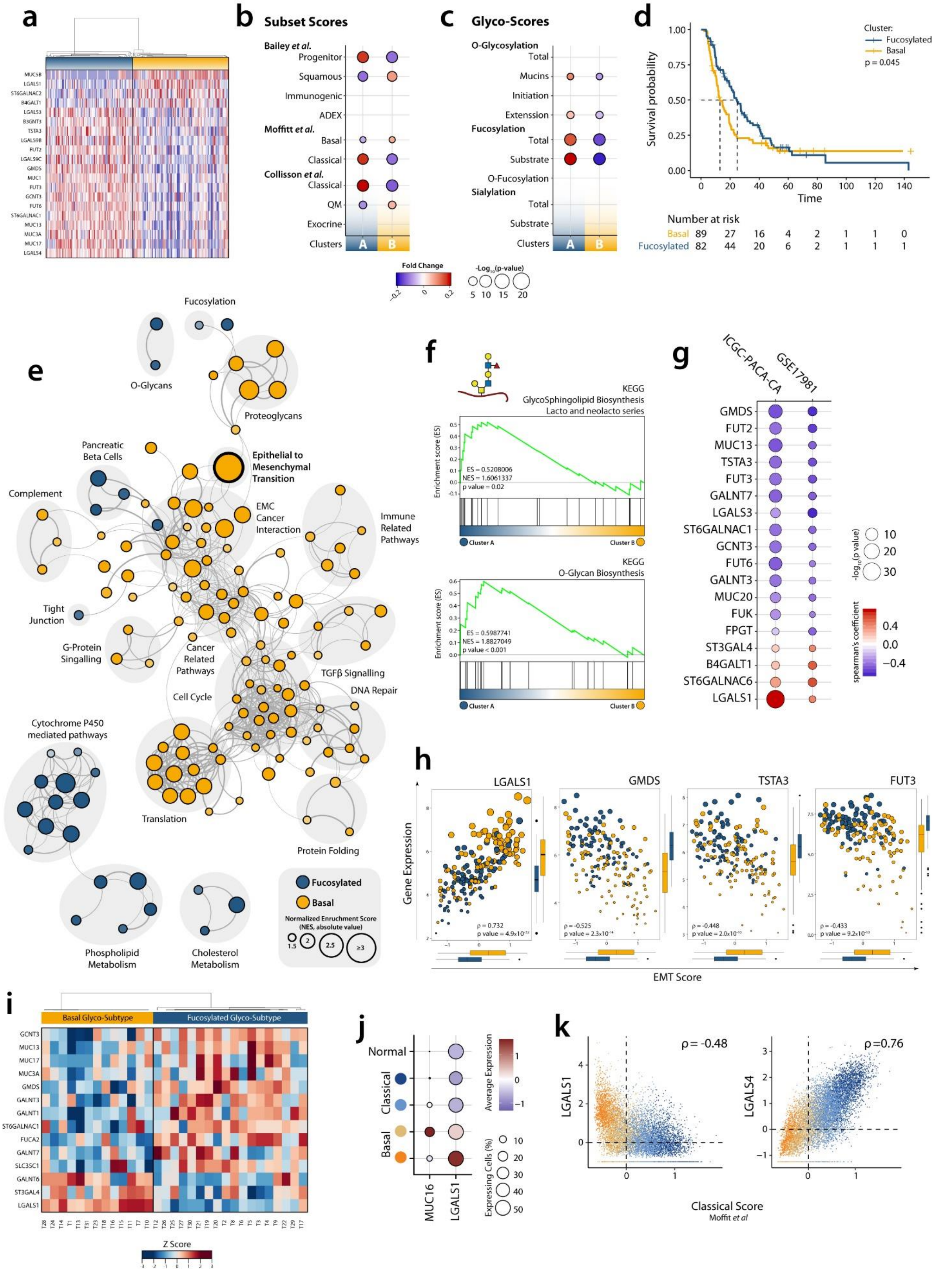

Figure S4. Identification and Characterization of glycosylation subtypes in transcriptomic data from micro-dissected tissue (ICGC-PACA-CA). Related to Figure 3. a) Heatmap of differential expressed glyco-code related genes. b) GSVA enrichment score for gene sets associated with subtypes previously described and c) glycosylation pathways. d) Survival analysis of Fucosylated and Basal subtypes in ICGC-PACA-CA dataset. e) EnrichmentMap illustrating gene set

enriched in Fucosylated and Basal subtypes based on KEGG, Reactome and Hallmark gene set. (**F**) GSVA representation of the gene sets ‘KEGG Glycosphingolipid Biosynthesis – Lacto and neolacto series’* and ‘KEGG O-Glycan Biosynthesis’. g) Correlation of glyco-code related genes with EMT Score in two datasets: ICGC-PACA-CA and GSE17981. h) Correlation plots of different glyco-code related genes with EMT Score. i) Heatmap of glyco-code related genes in transcriptomic data from organoids. j) Expression of *LGALS1* and *MUC16* in the different tumour clusters in scRNA-Seq. k) Correlation of the expression of LGALS1 and LGALS4 with a Classical Score in tumour cells identified in scRNA-Seq. * ‘KEGG Glycosphingolipid Biosynthesis – Lacto and neolacto series’ includes genes associated with the extension and fucosylation of glycan structures.

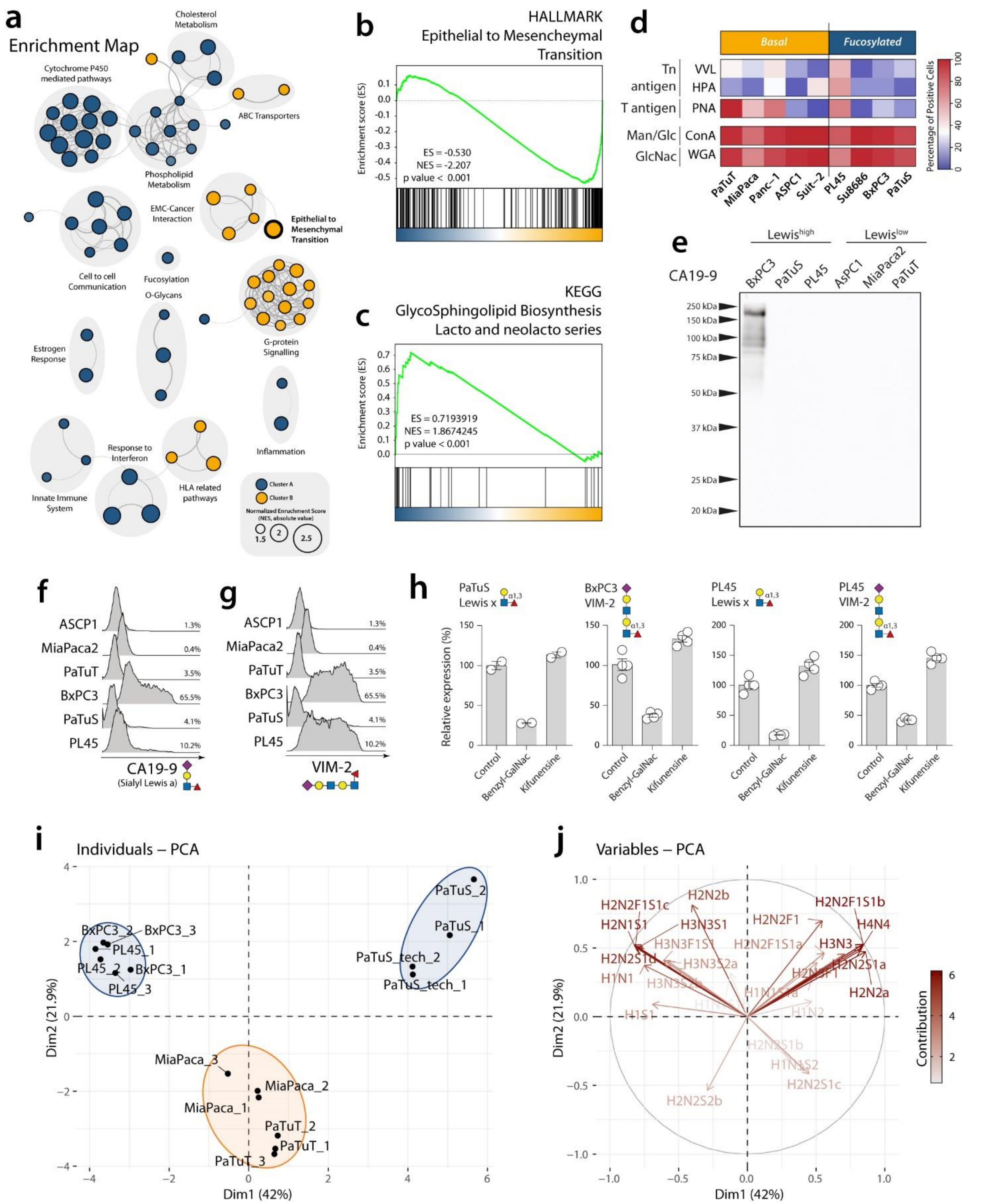

Figure S5. Characterization of Glycosylation different between subtypes of cell lines. Related to Figure 4. a) Enrichment map of subtypes in pancreatic cancer cell lines. b) GSVA representation of the genesets 'HALLMARK Epithelial to Mesenchymal transition' and c) 'KEGG Glycosphingolipid Biosynthesis – Lacto and neolacto series'. d) Heatmap representing the expression of different Lewis antigens in pancreatic cancer cell lines. Expression of the fucosylated antigens: CA19-9 (e,f) and VIM-2 (g). h) Inhibition of O and N- linked glycosylation pathways and evaluation of fucosylated antigens by flow cytometry using antibodies. Mean of Control was set as 100%. Statistical analysis of pairwise of each condition against control using One-way ANOVA with Dunnett method for multiple test correction (*p ≤ 0.05, **p ≤ 0.01). i) Principal component analysis (PCA) on relative abundance of the different O-glycans identified.

Subtypes are indicated by circles (Yellow, Basal; Blue, Fucosylated). j) Contribution of the different O-glycan structures identified to the PCA analysis.

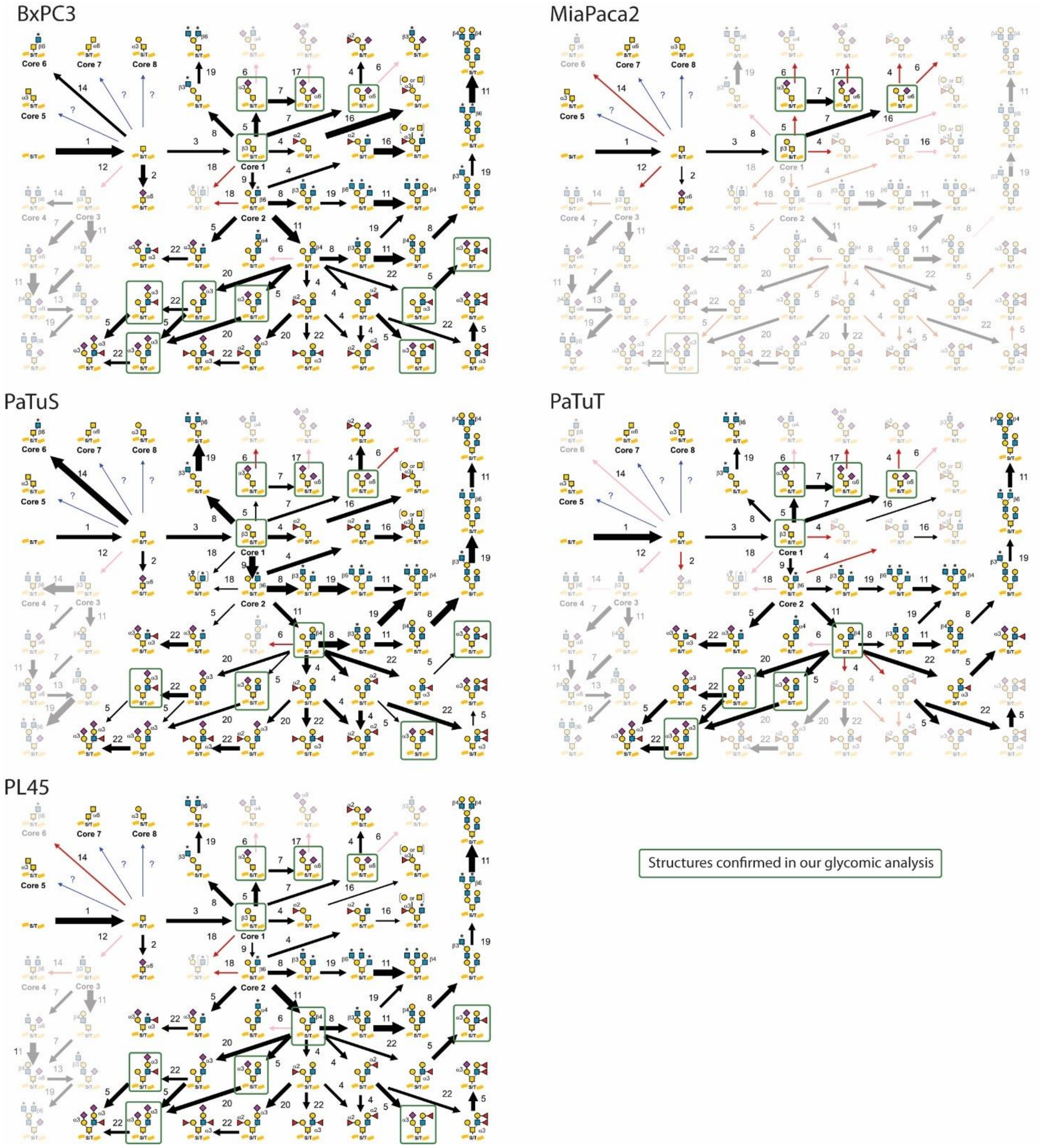

Figure S6. Prediction of O-Glycosylation pathway based on cell line gene expression using GlycoMaple.

Western Blots - Figure 4

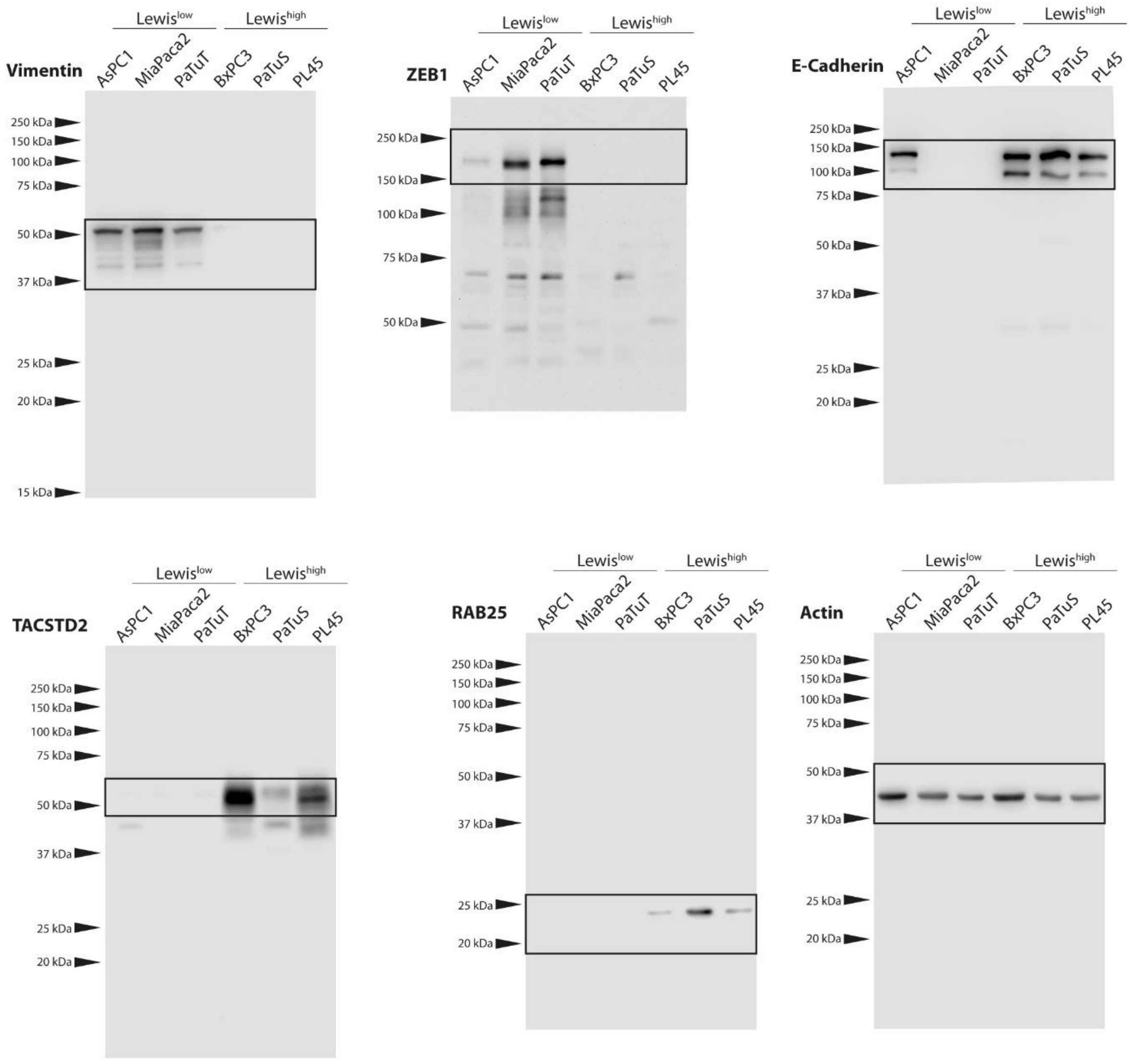

Western Blots - Figure 5

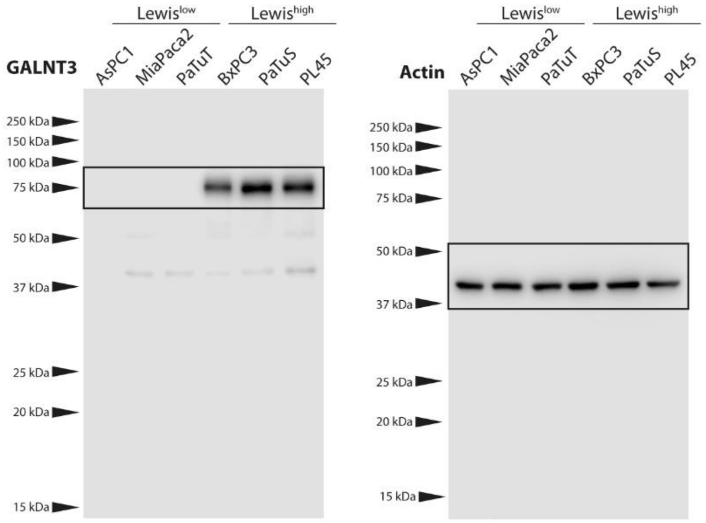

Figure S7. Western Blots. Related to Figure 4 and Figure 5.

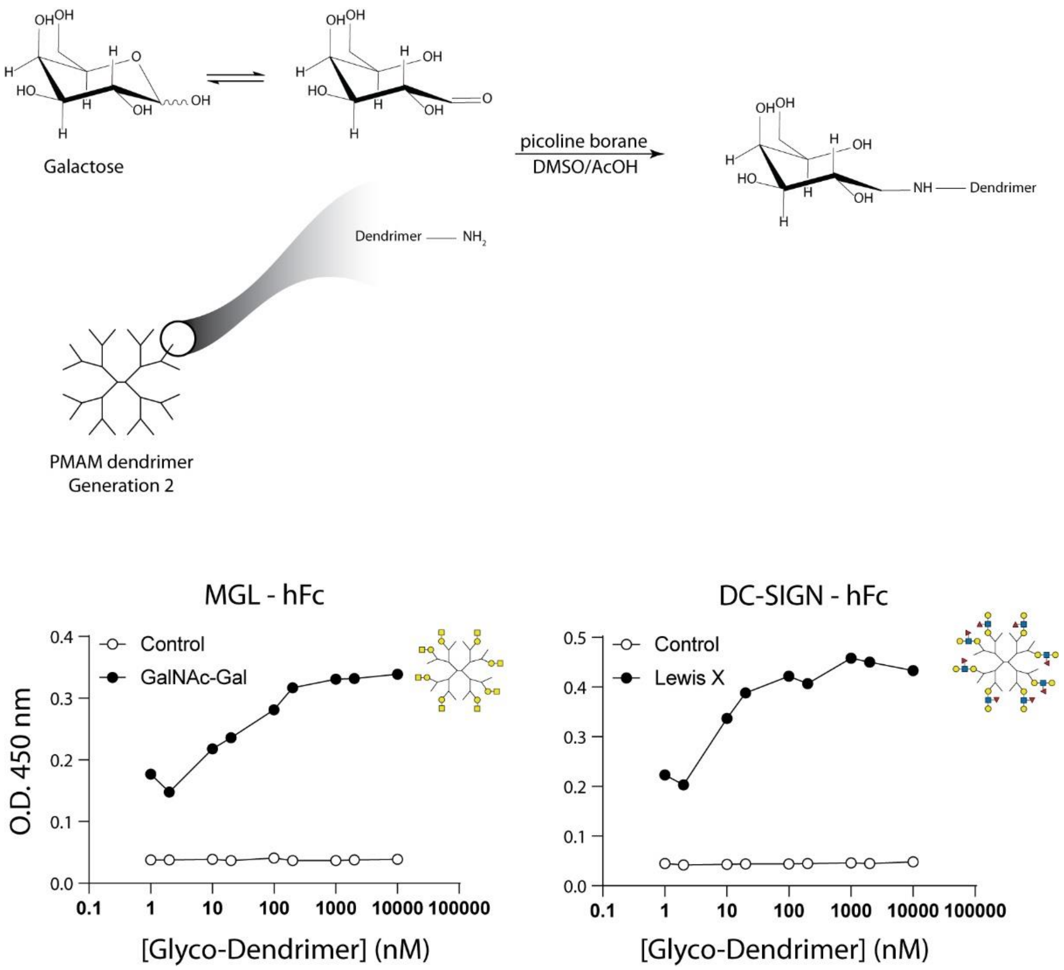

Figure S8. Synthesis and characterization of glycodendrimers. Scheme describing the synthesis of glyco-dendrimers by reductive amination (Top). For simplicity, we illustrated the synthesis using Galactosa, but the same principle applies for the rest of the glycan structures attached. We characterized the dendrimers by measuring their recognition by lectin receptors using CLR-hFc chimeric proteins in an ELISA-like assay.

Table S1. List of Glyco-code related genes.

**O-Glycosylation**			**Fucosylation**		**Sialylation**		**Galectins**
Enzymes Initiation	*Mucines*	*Enzymes Extension*	*Substrate Synthesis*	*Fucosyl-transferase*	*Substrate Synthesis*	*Sialyl-transferase*	
GALNT1	*MUC1*	*C1GALT1*	*FKGP*	*FUT1*	*CMAS*	*ST3GAL1*	*LGALS1*
GALNT2	*MUC2*	*C1GALT1C1*	*FPGT*	*FUT2*	*GNE*	*ST3GAL2*	*LGALS2*
GALNT3	*MUC3A*	*GCNT1*	*FUK*	*FUT3*	*NANP*	*ST3GAL3*	*LGALS3*
GALNT4	*MUC3B*	*GCNT2*	*GMDS*	*FUT4*	*NANS*	*ST3GAL4*	*LGALS4*
GALNT5	*MUC4*	*GCNT3*	*SLC35C1*	*FUT5*	*SLC35A1*	*ST3GAL5*	*LGALS7*
GALNT6	*MUC5AC*	*GCNT4*	*TSTA3*	*FUT6*		*ST3GAL6*	*LGALS7B*
GALNT7	*MUC5B*	*GCNT6*		*FUT7*		*ST6GAL1*	*LGALS8*
GALNT8	*MUC6*	*B3GNT1*	*Fucosidase*	*FUT8*	*Sialidases*	*ST6GAL2*	*LGALS9*
GALNT9	*MUC7*	*B3GNT2*	*FUCA1*	*FUT9*	*NEU1*	*ST6GALNAC1*	*LGALS9B*
GALNT10	*MUC8*	*B3GNT3*	*FUCA2*	*FUT10*	*NEU2*	*ST6GALNAC2*	*LGALS9C*
GALNT11	*MUC9*	*B3GNT4*		*FUT11*	*NEU3*	*ST6GALNAC3*	*LGALS12*
GALNT12	*MUC10*	*B3GNT5*		*POFUT2*	*NEU4*	*ST6GALNAC4*	*LGALS13*
GALNT13	*MUC11*	*B3GNT6*		*POFUT1*		*ST6GALNAC5*	*LGALS14*
GALNT14	*MUC12*	*B3GNT7*				*ST6GALNAC6*	*LGALS16*
	MUC13	*B3GNT8*				*ST8SIA1*	
	MUC15	*B3GNT9*				*ST8SIA2*	
	MUC16	*B3GNTL1*				*ST8SIA3*	
	MUC17	*B4GALT1*				*ST8SIA4*	
	MUC19	*B4GALT2*				*ST8SIA5*	
	MUC20	*B4GALT3*				*ST8SIA6*	
	MUCL1	*B4GALT4*					
		B4GALT5					
		B3GALT1					
		B3GALT2					
		B3GALT4					
		B3GALT5					
		B3GALT6					

Table S2. Characteristics of the datasets used in this paper.

**Differential gene expression**<br>Normal vs Tumor Tissue			
GSE ID	*n **	*Platform*	*Reference*
GSE15471	36 T / 36 NA	Microarray - Affymetrix Human Genome U133 Plus 2.0 Array	Badea, L. *et al* (2008)
GSE16515	36 T / 16 N	Microarray - Affymetrix Human Genome U133 Plus 2.0 Array	Pei, H. *et al* (2009)
GSE62452	69 T / 61 N	Microarray - Affymetrix Human Gene 1.0 ST Array	Shen, J. *et al* (2015)
GSE71729	145 T / 49 N	Microarray - Agilent - 014850 Whole Human Genome Microarray 4x44K G4112F	Moffitt, R.A. *et al* (2015)

**Clustering & Characterization**			
Study	*n***	*Platform*	*Reference*
TCGA PAAD	150	RNA-Seq - Illumina HiSeq 2000	Raphael, *et al* (2017)
E-MTAB-6134	309	Microarray - Affymetrix Human Genome U219 Array	Puleo, F. *et al* (2018)
ICGC – PACA – AU	269	Microarray - Illumina HumanHT-12 v4 Expression BeadChips	Bailey, P. *et al* (2016)
E-MTAB-6830	90	RNA-Seq - Illumina HiSeq2500	Dijk, F. et al (2020)
ICGC – PACA – CA***	234	RNA-Seq - Illumina HiSeq 2500	Connor, A. A. *et al* (2019)

* N = Normal Tissue; T = Tumor Tissue; ** All Tumor samples; *** Micro-dissected samples.

Table S3. Glyco-Subtypes in pancreatic cancer cell lines. Related to Figure 3.

1 = ***Basal*** Subtype
2 = ***Fucosylated*** Subtype

Cell line	Cluster
ASPC1	1
BXPC3	2
CAPAN1	2
CAPAN2	2
CFPAC1	1
DANG	1
HPAC	2
HPAFII	2
HS766T	1
HUPT3	2
HUPT4	2
KCIMOH1	2
KLM1	2
KP2	1
KP3	1
KP4	1
MIAPACA2	1
PANC0203	2
PANC0327	2
PANC0403	2
PANC0504	2
PANC0813	2
PANC1	1
PATU8902	1
PATU8988S	2
PATU8988T	1
PK45H	1
PK45P	1
PK59	1
PK8	2
PL45	2
PSN1	1
QGP1	1
SU8686	2
SUIT2	1
SW1990	1
TCCPAN2	1
YAPC	1
