# Supplementary material for: Analysis of the glyco-code in pancreatic ductal adenocarcinoma identifies glycan-mediated immune regulatory circuits
Source: Commun Biol. 2022 Jan 11;5:41. doi: 10.1038/s42003-021-02934-0 (PMC8752754; doi:10.1038/s42003-021-02934-0)
Supplement: Supplementary file 3 — Description of Additional Supplementary Files [file 42003_2021_2934_MOESM3_ESM.pdf]

Description of Additional Supplementary Files

File name: Supplementary Data
Description: Raw data of the experimental results shown in the manuscript.
