# Supplementary material for: Analysis of the glyco-code in pancreatic ductal adenocarcinoma identifies glycan-mediated immune regulatory circuits
Source: Commun Biol. 2022 Jan 11;5:41. doi: 10.1038/s42003-021-02934-0 (PMC8752754; doi:10.1038/s42003-021-02934-0)
Supplement: Supplementary file 5 — Reporting Summary [file 42003_2021_2934_MOESM5_ESM.pdf]

nature research

Corresponding author(s): Dr. Yvette van Kooyk

Last updated by author(s): Sep 14, 2021

Reporting Summary

Nature Research wishes to improve the reproducibility of the work that we publish. This form provides structure for consistency and transparency in reporting. For further information on Nature Research policies, see our Editorial Policies and the Editorial Policy Checklist.

Statistics

For all statistical analyses, confirm that the following items are present in the figure legend, table legend, main text, or Methods section.

n/a	Confirmed	
☐	☒	The exact sample size (n) for each experimental group/condition, given as a discrete number and unit of measurement
☐	☒	A statement on whether measurements were taken from distinct samples or whether the same sample was measured repeatedly
☐	☒	The statistical test(s) used AND whether they are one- or two-sided<br>*Only common tests should be described solely by name; describe more complex techniques in the Methods section.*
☒	☐	A description of all covariates tested
☐	☒	A description of any assumptions or corrections, such as tests of normality and adjustment for multiple comparisons
☐	☒	A full description of the statistical parameters including central tendency (e.g. means) or other basic estimates (e.g. regression coefficient) AND variation (e.g. standard deviation) or associated estimates of uncertainty (e.g. confidence intervals)
☐	☒	For null hypothesis testing, the test statistic (e.g. F, t, r) with confidence intervals, effect sizes, degrees of freedom and P value noted<br>*Give P values as exact values whenever suitable.*
☒	☐	For Bayesian analysis, information on the choice of priors and Markov chain Monte Carlo settings
☐	☒	For hierarchical and complex designs, identification of the appropriate level for tests and full reporting of outcomes
☒	☐	Estimates of effect sizes (e.g. Cohen's d, Pearson's r), indicating how they were calculated

Our web collection on statistics for biologists contains articles on many of the points above.

Software and code

Policy information about availability of computer code

Data collection	BD FACSDiva™, Summit™, LAS X (Version 3.3.0.16799)
Data analysis	Cytoscape (Version 3.6.1), FlowJo X, R (Version 3.5.1), RStudio (Version 1.1.453), GraphPad Prism 7.0, Microsoft Office Professional Plus 2016. Transcriptomic data was performed in RStudio using the following packages: limma (Version 3.45.7), GSVA (Version 1.37.2), NicheNet, survminer (Version 0.4.7), Seurat (Version 3.2.2)

For manuscripts utilizing custom algorithms or software that are central to the research but not yet described in published literature, software must be made available to editors and reviewers. We strongly encourage code deposition in a community repository (e.g. GitHub). See the Nature Research guidelines for submitting code & software for further information.

Data

Policy information about availability of data

All manuscripts must include a data availability statement. This statement should provide the following information, where applicable:

- Accession codes, unique identifiers, or web links for publicly available datasets
- A list of figures that have associated raw data
- A description of any restrictions on data availability

Transcriptomic data was downloaded from the NCBI GEO database (https://www.ncbi.nlm.nih.gov/geo/) with the following accession numbers: GSE1547117, GSE1651518, GSE6245219, GSE7172920. The single cell RNA-Seq data was downloaded from the Genome Sequence Archive (https://bigd.big.ac.cn/gsa/) under the project PRJCA00106358. The data from the PAAD project of the TCGA is available for download from the Broad Institute GDAC Firehose (https://gdac.broadinstitut.org)28. The transcriptomic data published by Puleo et al was downloaded from the ArrayExpress database with the accession number E-MTAB-613426. Data from the PACA-AU project of the ICGC (release 25) was obtained from https://dcc.icgc.org/27. The remaining data are available within the article, supplementary information or available from the authors upon request.

Field-specific reporting

Please select the one below that is the best fit for your research. If you are not sure, read the appropriate sections before making your selection.

☒ Life sciences ☐ Behavioural & social sciences ☐ Ecological, evolutionary & environmental sciences

For a reference copy of the document with all sections, see nature.com/documents/nr-reporting-summary-flat.pdf

Life sciences study design

All studies must disclose on these points even when the disclosure is negative.

Sample size	No statistical method was used to pre-determine sample size. In the experiments where human monocytes or macrophages were used, at least cells derived from 4 healty donors was used: Co-Culture of macrophages with cell lines n=4, Dendrimer stimulation of macrophages n = 7.
Data exclusions	Data was excluded from analysis only if positive controls did not work properly, as it could be for the standard curve in a cytokine ELISAs.
Replication	Each experiment performed in this paper has been successfully repeated independently at least 3 times.
Randomization	No randomization was used.
Blinding	Researchers were not blinded when conducting analysis of samples.

Reporting for specific materials, systems and methods

We require information from authors about some types of materials, experimental systems and methods used in many studies. Here, indicate whether each material, system or method listed is relevant to your study. If you are not sure if a list item applies to your research, read the appropriate section before selecting a response.

Materials & experimental systems

n/a	Involved in the study	
☐	☒	Antibodies
☐	☒	Eukaryotic cell lines
☒	☐	Palaeontology and archaeology
☒	☐	Animals and other organisms
☐	☒	Human research participants
☒	☐	Clinical data
☒	☐	Dual use research of concern

Methods

n/a	Involved in the study	
☒	☐	ChIP-seq
☐	☒	Flow cytometry
☒	☐	MRI-based neuroimaging

Antibodies

Antibodies used

AJoJo1
##Flow Cytometry and Microscopy##
Anti-PanCytokeratine - AlexaFluor 488
Anti-CD163 - Brilliant Violet 421™
Anti-Alpha-Smooth Muscle Actin - eFluor® 570
Anti-DC-SIGN (AZN-D1) - AlexaFluor 488
Anti-HLA-DR - BV786
Anti-E-Cadherin - AlexaFluor 647
Anti-Vimentin - AlexaFluor 488
Goat anti rabbit IgG - AlexaFluor 488
Anti LewisY antibody
Anti LewisX antibody
Anti-mouse IgM - FITC
Anti-mouse IgG - FITC
Anti-human IgG - FITC

##Western Blot##
CA19-9 Antibody (SPM110)
Purified anti-human E-Cadherin (24E10)
Purified anti-human Vimentin
Purified anti-human TMEM30B
Purified anti-human MAL2
Purified anti-human Rab25

	Purified anti-human GALNT3 Antibody<br>Polyclonal anti-human ZEB1<br>Polyclonal anti-human ZEB2<br>Purified anti-β-actin Antibody<br>Goat anti-rabbit - HRP<br>Rabbit anti-mouse - HRP<br>Goat anti-sheep - HRP<br><br>##ELISA##<br>Capture Antibody - IL-10<br>Detection Antibody - IL-10<br>Capture Antibody - IL-6<br>Detection Antibody - IL-6<br>Capture Antibody - TNFa<br>Detection Antibody - TNFa
Validation	Statements in antibodies data sheets provided by manufacturer. All antibodies were optimized and validated by serial dilutions.

Eukaryotic cell lines

Policy information about cell lines

Cell line source(s)	ASPC1, Mia PaCa-2 and PL45 were acquired from ATCC. BxPC3 are a kind gift from Dr. A. Frampton (Imperial College, London, UK). PaTuS and PaTuT are a kind gift from Dr. I. van Die (Amsterdam UMC, The Netherlands).
Authentication	Cell lines were tested were tested for their authentication by STR-PCR, performed by BaseClear (Leiden, The Netherlands), previous to the start of the project.
Mycoplasma contamination	Cell lines were routinely tested for Mycoplasma using PCR, resulting all negative.
Commonly misidentified lines<br>(See ICLAC register)	No Commonly misidentified cell lines were used in this paper.

Human research participants

Policy information about studies involving human research participants

Population characteristics	*Describe the covariate-relevant population characteristics of the human research participants (e.g. age, gender, genotypic information, past and current diagnosis and treatment categories). If you filled out the behavioural & social sciences study design questions and have nothing to add here, write "See above."*
Recruitment	Biopsies were collected from patients that undergo to whipple surgery. Samples with a pathological assessment of PDAC were used. For the analysis of sialic acids in normal and tumor tissue, samples containing adjacent normal tissue were analyzed. No bias that could affect the results were identified.
Ethics oversight	Medical Ethical Committee from the Amsterdam UMC, Location VUmc.

Note that full information on the approval of the study protocol must also be provided in the manuscript.

Flow Cytometry

Plots

Confirm that:

☒ The axis labels state the marker and fluorochrome used (e.g. CD4-FITC).

☒ The axis scales are clearly visible. Include numbers along axes only for bottom left plot of group (a 'group' is an analysis of identical markers).

☒ All plots are contour plots with outliers or pseudocolor plots.

☒ A numerical value for number of cells or percentage (with statistics) is provided.

Methodology

Sample preparation	For the analysis of cell lines, cells were harvested using a mix of EDTA/Trypsin.
Instrument	CyAn™ ADP Analyzer or BD LSRFortessa™.
Software	Acquisition: BD FACSDiva™ for Fortessa, Summit™ Software for CyAn.<br>Analysis: FlowJo X.
Cell population abundance	*Describe the abundance of the relevant cell populations within post-sort fractions, providing details on the purity of the samples and how it was determined.*

Gating strategy

For the determination of glycan structures in PDAC cell lines, only single live cells were analyzed by gating in Viability Dye negative cells.

☐ Tick this box to confirm that a figure exemplifying the gating strategy is provided in the Supplementary Information.
